# Influenza A Pandemic (H1N1) 2009 Virus and HIV

**DOI:** 10.3201/eid1607.091339

**Published:** 2010-07

**Authors:** Marta Mora, Elena Rodriguez-Castellano, José Ramón Pano-Pardo, Juan González-García, Carolina Navarro, Juan Carlos Figueira, José Ramón Arribas

**Affiliations:** Author affiliation: Hospital Universitario La Paz, Madrid, Spain

**Keywords:** Influenza A virus, H1N1 subtype, HIV, influenza, viruses, human, letter

**To the Editor:** The effects of influenza A pandemic (H1N1) 2009 virus infection in HIV-infected patients are unknown. We describe an HIV-infected patient with severe pandemic (H1N1) 2009 virus infection.

The patient was a 37-year-old, HIV-positive, former intravenous drug user in a methadone-substitution program. She had a history of smoking, hepatitis C, and mild chronic obstructive pulmonary disease not requiring treatment. Since 2007 her viral load had been <50 HIV RNA copies/mL, for which she received tenofovir, emtricitabine, and lopinavir/ritonavir. Her CD4 count in March 2009 was 542 cells/µL (25%).

On June 24, 2009, the patient entered the Hospital Universitario La Paz after 3 days of dyspnea and fever, without cough or sputum. Temperature was 39°C, blood pressure 118/74 mm Hg, pulse rate 110 beats per minute, respiratory rate 30 breaths per minute, and oxygen saturation 85% on room air (fraction of inspired oxygen [FiO_2_] 21%). Lung wheezes were audible. Laboratory testing showed leukocytosis with neutrophilia and oxygen partial pressure (pO_2_) 70.9 mm Hg (FiO_2_ 21%). Chest radiograph findings were consistent with bacterial pneumonia (Figure, panel A). Empirical treatment with clarithromycin and ceftriaxone was started. After full clinical recovery, the patient was discharged on June 30 and prescribed oral clarithromycin and cefixime.

**Figure:**
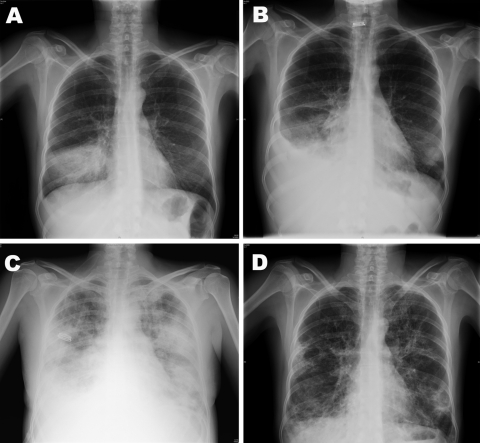
Chest radiographs of 37-year-old, HIV-positive woman with severe pandemic (H1N1) 2009 virus infection, 2009. A) June 24, alveolar infiltrate in the right lower lobe. B) July 3, minimal pleural effusion, alveolar infiltrate on right lower lobe, and possibly left lower lobe infiltrate. C) July 6, bilateral alveolo-intertitial infiltrates. D) July 29, bilateral peribroncovascular thickening with fibro-cicatricial changes; conserved lung volumes.

At a routine follow-up visit on July 2, the woman was asymptomatic and had fewer leukocytes and neutrophils, creatine kinase 800 U/L (reference <145), and lactate dehydrogenase 373 U/L (reference <247). On July 3, she returned to the hospital because of dyspnea and high fever (38.5°C). Oxygen saturation was 75%, pO_2_ 33.8 mm Hg (FiO_2_ 21%), and blood pressure 90/54 mm Hg. Chest radiographs showed alveolar infiltrates in the right lower lobe (Figure, panel B). Because her deterioration was attributed to nosocomial infection, she was given meropenem, linezolid, and levofloxacine and was hospitalized. Within the next 96 hours, her condition deteriorated further to drowsiness, hypotension, oxygen saturation 92%, pO_2_ 60.5 mm Hg (FiO_2_ 50%), and new radiographic bilateral alveolo-interstitial infiltrates appeared (Figure, panel C).

On July 7, real-time reverse transcription–PCR of a nasopharyngeal swab confirmed influenza A pandemic (H1N1) 2009 virus (*1*). Other infectious causes for bacterial and viral pneumonia were excluded. The patient received mechanical ventilation for 7 days and oseltamivir. She was discharged after 21 days. She still had dyspnea after exertion and radiologic sequelae on chest radiograph (Figure, panel D).

No patients or healthcare workers who had had contact with the patient had confirmed pandemic (H1N1) 2009 virus infection. The patient shared a room with a highly immunocompromised HIV patient, who was negative for pandemic (H1N1) 2009 virus but received oseltamivir as prophylaxis.

Few data are available on pandemic (H1N1) 2009 virus infection in immunocompromised patients. Of 30 patients with pandemic (H1N1) 2009 in California (*2*), 6 had underlying conditions involving immunosuppression but none was infected with HIV. The clinical course of pandemic (H1N1) 2009 virus infection in immunocompromised patients was similar to that in nonimmunocompromised patients, although not all received oseltamivir. The patient reported here was not severely inmunosuppresed; her CD4 count was stable at >300 cells/μL.

Severe pandemic (H1N1) 2009 virus infection most commonly produces fever, dyspnea, respiratory distress, and bilateral patchy pneumonia (*3*), which can initially be interpreted as bacterial pneumonia and consequently treated with antimicrobial drugs (*2**–**7*). We believe that the patient reported here first had a community-acquired pneumonia with a rapid response to treatment and that she secondarily had respiratory distress caused by pandemic (H1N1) 2009 virus pneumonia. We cannot confirm whether the pandemic (H1N1) 2009 virus infection was nosocomial or community acquired.

The increased creatine kinase and lactate dehydrogenase and lymphopenia in this patient resemble that reported in Mexico by Perez-Padilla et al. (*3*). Although these laboratory parameters are unspecific, they could serve as an alert to pneumonia caused by pandemic (H1N1) 2009 virus instead of bacteria. The Centers for Disease Control and Prevention (CDC) recommends testing all HIV-infected patients suspected of having pandemic (H1N1) 2009 virus infection (*8*). The Spanish Ministry of Health and Madrid Department of Health recommends this testing for patients with an erratic outcome from common pneumonia.

For HIV-infected patients who meet case definitions for confirmed, probable, or suspected pandemic (H1N1) 2009 virus infection, CDC recommends empiric antiviral drug treatment (*8*). There are no known contraindications for co-administration of oseltamivir, a neuraminidase inhibitor, with antiretroviral medications; no interactions have been demonstrated (*8**,**9*).

Although clinical signs and treatment of pandemic (H1N1) 2009 are similar for patients with and without HIV infection, HIV-infected patients with suspected pandemic (H1N1) 2009 virus symptoms should be treated as soon as possible. CDC recommends the use of influenza antiviral drugs, but this recommendation might change as additional data on this therapy for HIV-infected patients become available. At this time, however, we strongly recommend use of antiviral drugs for HIV-infected patients with suspected pandemic (H1N1) 2009 virus.
